# Supplementary material for: Defects in Glutathione System in an Animal Model of Amyotrophic Lateral Sclerosis
Source: Antioxidants (Basel). 2023 Apr 27;12(5):1014. doi: 10.3390/antiox12051014 (PMC10215445; doi:10.3390/antiox12051014)
Supplement: Supplementary file 1 [file antioxidants-12-01014-s001.zip › antioxidants-2324176-supplementary.pdf]

Supplementary Figure S1

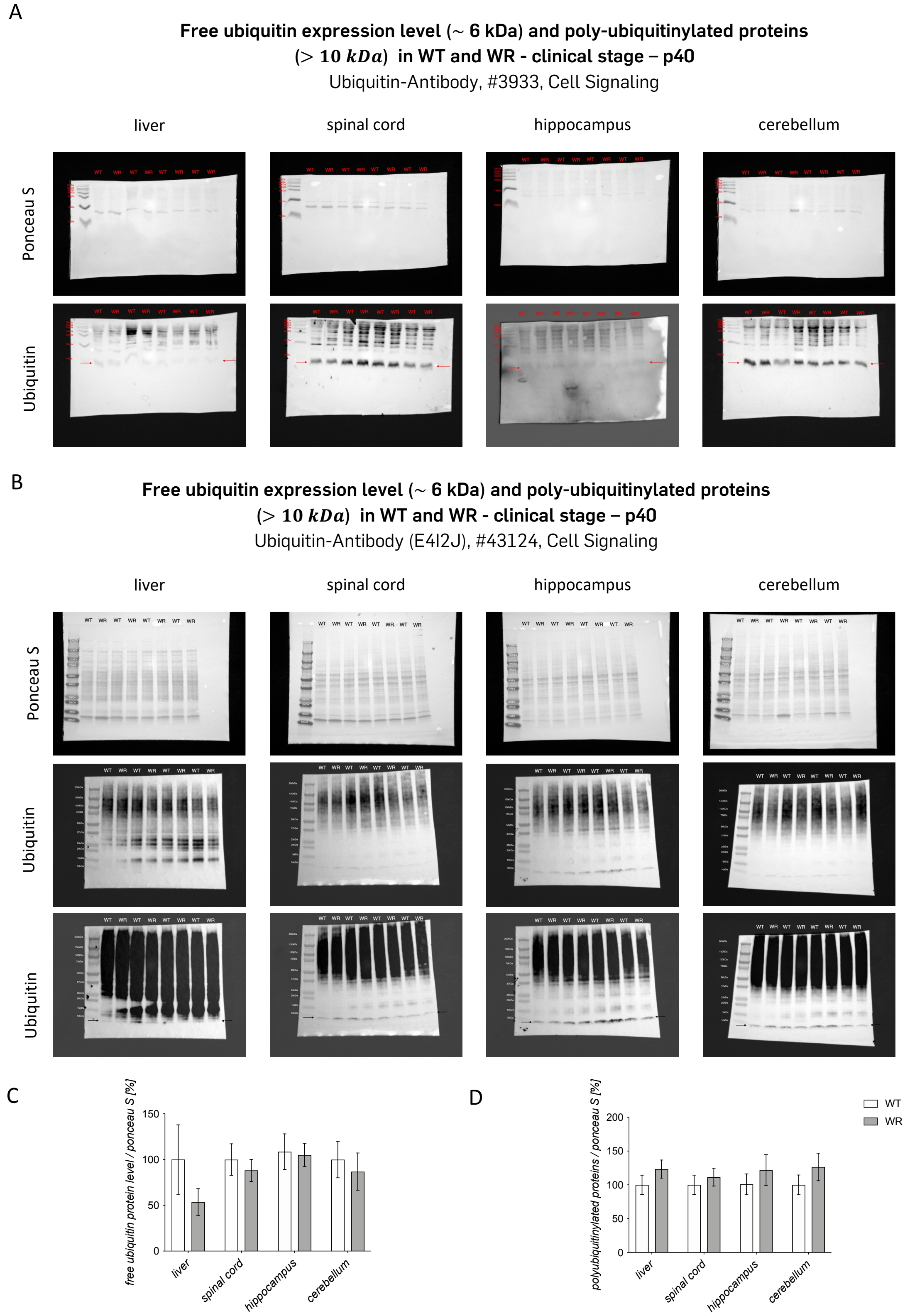

Figure S1 No changes in the ubiquitinylation of proteins in different tissues of the p40 wobbler mouse. To visualize free ubiquitin and poly-ubiquitinylated proteins, 20 µg of protein lysate from the liver, spinal cord, hippocampus and cerebellum were applied to an SDS gel. A western blot was performed, and ubiquitin was detected using two different antibodies. **A** shows the results of immunodetection using antibody #3933, Cell Signaling. **B** shows the results of immunodetection using antibody #43124, Cell Signaling. Ponceau S staining was performed to normalize the ubiquitin values. Free ubiquitin was identified at a size of about 6 kDa. All signals larger than 15 kDa were used for the analysis of poly-ubiquitinylated proteins. **C** shows the normalized results of both experiments of the free ubiquitin (marked with arrows in the blots). **D** shows the results of both experiments of the poly-ubiquitinylated proteins. N=4.
